# The Invasive *Ailanthus altissima*: A Biology, Ecology, and Control Review

**DOI:** 10.3390/plants13070931

**Published:** 2024-03-23

**Authors:** Jordi Soler, Jordi Izquierdo

**Affiliations:** Department of Agri-Food Engineering and Biotechnology, Universitat Politècnica de Catalunya, 08860 Castelldefels, Spain; jordi.izquierdo@upc.edu

**Keywords:** Tree of Heaven, biological invasion, germination requirements, biological control, chemical control, management

## Abstract

Tree of Heaven (*Ailanthus altissima* (Mill.) Swingle) is a tree native to China which has invaded disturbed areas in many regions worldwide. Its presence endangers natural ecosystems by displacing native species, modifying habitats, changing community structures, and affecting ecosystem processes. Its invasive nature is enhanced by its high ability to reproduce both vegetatively through root regrowth and sexually through seeds. Seeds, which are wind dispersed, are the main mechanism by which this species reaches new habitats. When they germinate and develop the root system, roots emit new shoots that contribute to a rapid increase in the tree density and the subsequent expansion of the population nearby. The contradictory results about the ecological requirements for seeds to germinate and their degree of dormancy and longevity indicate the complexity and difficulty of understanding the mechanisms that govern the biology and adaptability of this plant. The management of this weed aims at its eradication, with programs based on herbicide applications carried out by injecting the active ingredient directly to the trunk. But, not many active ingredients have shown total control, so new ones should be tested in order to increase the range of available herbicides. During the last few decades, some biological agents have been identified, but their efficacy in controlling the tree and their safety for the local flora have not yet been determined. A correct management strategy should take into account all these aspects in order to contain the expansion of this species and, ultimately, allow its eradication.

## 1. Introduction

Tree of Heaven (*Ailanthus altissima* (Mill.) Swingle is one of the most invasive weeds in temperate climates of the world [1]. It is a dioecious species that belongs to the family Simaroubaceae and to the genus *Ailanthus*, which comprises up to 15 different species, as found in the extensive review by Kowarik [2]. Adults can reach a height of up to 27 m and 1 m diameter at breast height [3]. The leaves are sparse and pinnaticomposite and they can be glabrous or pubescent, with leaf length measuring up to 90 cm [4]. The cotyledons are rounded and with epigeal germination [5]. Male flowers contain 10 stamens, while female flowers have 10 non-functional stamens and a pistil with 5–6 free carpels. The fruits are winged and elongated up to 5 cm in length, samara type, and grouped in leafy and hanging clusters [4]. The blooming period in Mediterranean latitudes occurs during May and July [6]. The diameter at breast height is a good indicator of the amount of seed production [7], with a female adult tree producing up to 300,000 seeds per year [8].

Seeds are mainly dispersed by wind [9], but they can remain in the canopy throughout the winter [5]. The species has a root system with high potential to expand by producing new shoots that contribute to its vegetative dispersion. Most of the roots are found in the first 46 cm of soil [3], with roots sprouts found up to 27 m from the parent tree [10]. More information about the biology of this species can be found in [11].

*A. altissima* is present in all continents except Antarctica and prefers areas with high human disturbance [2]. The initial ornamental use of this tree in many gardens and backyards favored its spread; however, because of its invasive traits, ornamental uses have decreased, and the origin of new infestations nowadays lies with existing natural populations [11]. Infestations seem to be dependent on the level of disturbance of the invaded area [12], as a positive correlation is found between the presence of *A. altissima* along different parts of the Danube river and areas with dense human population [13]. Additionally, it is not affected by urban pollution [14]. It adapts well to a wide range of soils and prefers warm climes [3], although it is able to establish itself in many different climatic conditions [11]. It is well established in the Mediterranean basin, in which many eradication programs have been carried out [15]. 

As an invasive plant, *A. altissima* has all the detrimental ecological effects on local ecosystems that have been described for these types of species elsewhere [16,17]. However, it has several particular traits that contribute to generating further damage. The rapid entry into flowering (fourth year), the huge amount of seed production, the wind dispersal, and the vegetative reproduction favor its expansion and enhance its persistency [7]. Its ecological preference for altered and degraded ecosystems and its well-developed root system make this species frequent in communication corridors such as railways, freeways, or walkways [2], in which it damages constructions and pavements and reduces visibility [18], in rural areas along fencerows, woodland edges, or forest openings [2], and in heritage areas such as archaeological monuments, degrading them [19,20,21,22]. But, apart from physical damage, this species has allelopathic effects. Ailanthone, a quassinoid compound, has exhibited pre- and postemergence herbicidal activity against other species [11]. It can be found in different parts of the tree and negatively affects the growth of different native species [23], allowing an increase in the presence of other non-native species [24]. Assays with concentrated extracts of ailanthone have shown different effects towards different weeds [25], with dicots being the more affected species [26]. Perhaps this fact may explain why the removal of *A. altissima* in natural areas does not allow native plants to recover until two years later [27]. The modification of bacterial colonies from soil and retardations in the growth of plants under *A. altissima* canopies have also been reported [28]. 

## 2. Seed Dormancy

Although *A. altissima* was classified as a species having seeds with non-dormant embryos [29], some years later, dormancy was described [30,31]. The environmental requirements for *A. altissima* seeds to germinate have been shown to be greatly variable according to the bibliography, which suggests that several intrinsic factors, such as embryo immaturity or the presence of inhibitors, must have some influence on the level of dormancy of the seeds [32]. Additionally, the level of dormancy of seeds seems to also be related to the environmental conditions suffered by the parent plant, as great variability was found among the germination rates of seeds collected at the same time from different individuals [7]. In fact, the seeds have to break both the physiological dormancy produced by the presence of inhibiting hormones and the mechanical barrier of the coat that contains the embryo [33]. 

In order to break the dormancy of *A. altissima* seeds, different methods have been tested. Gibberellic acid [34,35], cold stratification [3,33,36,37,38,39,40,41], wet or dry stratifications [42,43], and sulfuric acid or boiling water [33] have been applied to the seeds over different periods of time. From the results reported, cold stratification seems to give the highest germination rates. A summary of these methods and the results obtained can be found in Table 1. 

## 3. Seed Germination

### 3.1. Temperature Requirements

The variability of the results found makes setting an optimal temperature for seed germination difficult. In growth chambers under constant temperature, the highest germination rates were obtained at 15 °C and the lowest at 30 °C [37]. Simulating different heat treatments, as forest fires would do, germination decreased with increasing heat temperatures [41]. However, with an alternating temperature regime of 15/6 °C, seeds germinated almost four times less than at an alternant higher temperature of 30/20 °C [39]. If the coat of the seed is removed, the best germinating temperatures for naked embryos were found by alternating 25/30 °C or by a constant 30 °C [40]. In growth chambers at 20.5 °C, germination reached 90 and 97% depending on the place the seeds were collected [44]. At a constant 20 °C, germination rates ranged from 44.4 to 26.2% depending on the intensity of the chamber flux light [43]. In greenhouse conditions with natural light and a temperature ranging between 21 and 24 °C, germination rates were 66.1% [45] or 90.7% and 91.4% depending on the natural stands from which the seeds were collected [46]. A pre-treatment with gibberellic acid showed 30 °C as the optimal temperature [34]. 

### 3.2. Light Requirement for Germination and Growth

Although *A. altissima* is considered to be a shade intolerant species [3], it can germinate and grow under a natural forest canopy with low light conditions [53] or be competitive in a closed-canopy forest [54]. Simulating different leaf litter layers over *A. altissima* seeds in the greenhouse, no differences in germination were found, meaning that a lack of direct light it is not the main condition for its germination [50]. Furthermore, measurements of leaf water potential found no differences between trees growing under high irradiance conditions with shaded ones. However, it seems that germination rates are affected by light exposure, because the average time needed for seedlings to emerge was longer when the flux of natural light was reduced, for example by using plastic nets to mimic shadow conditions [49] or positioning under a dense forest canopy [43]. The inhibition effect of the coat was also deduced from the study of [40], in which they found that naked embryos in dark conditions, which do not promote the germination of the seeds, were able to achieve a germination rate of 94%.

Additionally, longer photoperiods allowed seedlings to more quickly develop their vascular system than seedlings growing in completely dark conditions, although the increment was non-significant [55].

### 3.3. Water Requirements for Germination and Growth

In laboratory conditions and using Polyethylene glycol (PEG) to simulate different water stress conditions, the germination rate of *A. altissima* seeds decreased when reducing the water potential. Germination significantly decreased when water potential decreased from −2 to −4 bar, with almost no germination found at -6 bar and none at all at −8 bar [51,52]. Similar decreasing trends when lowering the water potential were observed by [56], although *A. altissima* seedlings supported better water stress than *Phytolacca americana* and *Robinia pseudoacacia* [57].

Greenhouse experiments on the effect of water availability on plant growth showed that decreasing irrigation regimes (1, 0.25, 0.1, and 0.05 L per week) reduced the leaf and root area of the plants, although the results were not statistically different [58]. With a similar water regime experiment (0.3 and 0.03 L per week) a positive correlation between drought and growth was found, with seedlings having a more reduced growth, height, and dry weight at low water availability [45]. The differences between both studies probably lie in the fact that the first experiment lasted two years while the second lasted only one, suggesting that plants have mechanisms to adapt to a water-scarce environment. In adult plants, the ability of this species to cope with drought may also be related to its ability to take water mainly from deeper soil layers (more than 75 cm) than from the first 25 cm of soil layers [59]. Other authors pointed out that *A. altissima* was more efficient in terms of root-to-leaf water transport capacity than native species [60].

Water exposure seems to affect seed germination. Some studies report increases in the germination rate of seeds exposed to 3 days of water (floating or submerged) compared to 20 days [48], although the opposite trend was found, with no descending rate observed for seeds floating for 5 months [47]. 

## 4. Seed Longevity 

For species that are reproduced by seeds, the longevity of the seeds is a key factor for determining the persistency of the species in the habitat. *A. altissima* seeds have very low level of predation [12,44] and although some authors found that the longevity of the *A. altissima* seed bank was not significant [8], or not enough to form a long-term seed bank [2,43], more recent studies have shown that the viability of stored seeds can be as long as three [37], five [38], or nine years [7].

## 5. Seedlings Survival

The survival of *A. altissima* seedlings depends primarily on soil water availability and the competition with the native flora [14]. If the native flora has a very dense canopy, seedlings will hardly survive in such a shady environment, but if the level of disturbance of the canopy forest is significant, more light will reach the understory, more seeds will germinate, and more seedlings will survive [37,43]. Shade and cold conditions act as limiting factors for seedlings’ establishment [11].

## 6. Dispersion

The main aim of any biological dispersal process is to allow the reproductive structures of the plant to reach long distances from the mother plant [48]. The distance is more related to the height of the plant than to the seed mass [54]. In the case of *A. altissima*, long distances from the seed source are mainly achieved by means of the wind [9,47,61] and secondarily by water [3] or animals such as birds or ants [54]. Wind is the primary source of dispersion as the fruit is a samara well-adapted to wind dispersal [3] and fruits may reach distances up to 200 m from the mother plant [62]. The final distance will depend on the wind speed and the orography, because some studies have found shorter distances [12,63,64]. Water is a secondary source of dispersion. Samaras are also adapted to float on water [48,65,66] and can be scattered downstream [47,54,67]. Seeds can remain viable in water for a long time (94.4% germinability after five months) [47]. 

## 7. Vegetative Reproduction

Asexual reproduction is an important trait to consider for this species, as new shoots from roots act as a dispersion mechanism [11]. The absence of a taproot is common [3] and a root system that has an asymmetrical shape, adapted to the soil characteristics [2]. New shoots can appear from stumps or roots, and the shapes of leaves vary if they appear from root sprouts (from unifoliolate to pentafoliolatemore or others) or seedlings (trifoliolate) [5]. When aerial parts suffer damage or die, new shoots from the root system appear [3].

## 8. Management

Different strategies such as chemical, mechanical, and biological or a combination of them have been applied for the management of *A. altissima* trees in natural ecosystems. Many of these strategies showed a good efficacy; however, it is important to point out that due to the high capacity of the plant to resprout from the root system as explained before, any actuation on well-established individuals will need long-term supervision to check the efficacy of the measurement [2,68,69], particularly when female trees are present. 

### 8.1. Mechanical

Mechanical control can be performed by hand or with any tool, but it is only effective against seedlings, because once the root system is established, cutting or breaking the roots will promote resprouting [5], and successive cuts will increase the number of shoots [11]. Mechanical control on established trees showed very weak control of the populations. For example, when comparing mechanical versus chemical, it was found a mortality of 21.3% by manual cutting versus near total control with different herbicides [70]. Other authors have demonstrated that herbicides such as glyphosate, imazapyr, picloram, triclopyr, or 2.4-D had better control than mechanical methods alone [21,71,72]. When comparing cutting versus herbicide application over the cut stump with glyphosate, imazapyr, or triclopyr, trees without herbicide produced more resprouts than trees with herbicide [73]. Similar results were obtained, with a mortality of 52% by only cutting compared to near 90%, when herbicides were applied over the cut stump [74]. A combination of mechanical actuations plus chemical control seems to be the best procedure rather than chemical control alone [68,71].

### 8.2. Chemical 

Herbicides are the most popular method to manage *A. altissima* populations [75]. Systemic herbicides are the most efficient particularly when applied at the end of the growing season because they are transported to the root system via the phloem with the descending movement of the sap [69]. 

The application of the herbicides on the trees is performed by means of different techniques: stem injection, basal bark, or cut stump. Stem injection is performed by making holes with a drill into the trunk and filling each hole with herbicide, by the E-Z-ject Lance system (injecting into the trunk solid capsules containing herbicide) or by hack-and-squirt which is spraying into cuts performed with a hack along the stem. Basal bark consists of spraying herbicides into the lower part of an uncut trunk. Cut stump consists of spraying or injecting the herbicide on the cut surface of the trunk. 

Herbicides have been applied diluted and undiluted, with different results. The best results with the stem injection technique were observed using undiluted glyphosate and making holes [76]. However, undiluted triclopyr applied by hack-and-squirt showed no total effectiveness over the trees [73]. The E-Z-ject Lance system has been tested with triclopyr and imazapyr [77,78], and glyphosate [71,78,79] with varied efficacy.

The efficacy of basal bark applications depended on the diameter of the tree. Diluted triclopyr showed good control in most cases [70,74,77,79], but diluted mixtures of triclopyr + fluroxypyr, aminopyralid + fluroxypyr or glyphosate alone did not show total mortality when applied to trees with bigger diameters [21]. 

For cut stump applications, mortality seems to depend on the concentration of the active ingredient. Spraying diluted active ingredients alone (i.e., triclopyr or glyphosate) or mixed (i.e., triclopyr + fluroxypyr, aminopyralid + fluroxypyr or glyphosate) did not achieve total mortality of the trees [21,73], while the same active ingredients undiluted reached total control [76]. Another case is granular herbicides like Metsulfuron methyl, where undiluted applications are not possible, which had a great mortality but not all trees died [74]. 

### 8.3. Biological

Although *A. altissima* tissues contain chemical compounds that likely act as a natural defense against pests [2], during the last decades many different organisms have been identified as biological agents of *A. altissima* trees, some of them with high specificity. These natural enemies are arthropods and fungus and most of them have been reported in Chinese ecosystems, although lately they have also been reported in the places where *A. altissima* has been introduced, probably due to accidental introductions [80]. Mites have been reported to attack leaves, with the genus *Aculus* spp. being the most mentioned ones [81,82,83,84]. Coleoptera such as *Eucryptorrhynchus brandti* and *Eucryptorrhynchus chinensis* have shown good specificity over *A. altissima* in China [80]. These coleoptera also showed good specificity in quarantine trials, preferring this tree over others when feeding at the larval stage and for oviposition [75,85,86] making them a good option for biological control. In Italy, the orange whitefly (*Leurocanthus spiniferus*) has been reported on *A. altissima* for the first time, but this insect cannot be considered a biological agent because the trees tolerated the infestation [87].

Some generalist insects have a range of hosts that include *A. altissima* leaves in their diet, such as the butterflies *Atteva punctella* and *Samia cynthia*, whose host range includes trees from the genus Simarouba [80,88] and the beetle *Maladera castanea* [3]. Regarding biological control, *A. altissima* acts as a host for the invasive pest *Lycorma delicatula* in North America [89].

Fungus has an important role in *A. altissima* biocontrol. Some *Verticilium*, *Alternaria,* and *Cercospora* species have shown good results as biocontrol agents [80]. The *Verticilium* species are the most important and they have been identified in many countries like the USA [3,90,91,92,93,94,95], Austria [96], Italy [97], and Spain [98]. Common symptoms of trees affected by *Verticillium* are premature defoliation, yellowish vascular discoloration, and final mortality [91]. Different strains of *Verticillium* may act depending on the climate, with *V. dahliae* being the most common in warm areas and *V. nonalfae* in cooler regions [96]. Trees can be infected during winter and show the first symptoms the next growing season. Under laboratory conditions, *V. nonalfalfae* was transmitted by *E. brandti*, which carried the propagules of the fungi externally and internally [99].

## 9. Challenges

*A. altissima* has a very extensive bibliography involving many other topics such as medicinal properties or phytosanitary activity of some of its components. All this information has not been cited in this review because it is not relative to the invasive aspect of this weed in natural ecosystems. However, from the information reviewed, it appears that there is a need for further study of the behavior of this prolific species. Understanding the mechanisms of seed dormancy, determining the ecological requirements for seeds to germinate, or finding the best herbicide combination to control this weed are some of the aspects that are not well known. Additionally, some challenges derived from its control still have to be addressed. The use of herbicides may provoke soil/water contamination for drift or root exudates. When managing its populations, the vegetal residues of the trees generated should be properly treated to avoid the negative effects of their allelopathic compounds, by converting the residues into mulching in a secure way. The biological control with the fungus *Verticillum* in the invaded areas faces the challenge of the possible effect on native flora. As this tree has been shown to have growth limitations when living in closed-canopy forests, it would be interesting to determine the best planting density of native species in order to deter its establishment. New research studies are needed in order to properly develop successfully management programs aimed to eradicate this weed from our natural systems.

## Figures and Tables

**Table 1:** Summary of germination requirements of *Ailanthus altissima* seeds according to different authors. * ≈ the number is an approximation from a graphic.

Seed Harvest Date	Germination Place	Pre-Treatment	Light/Dark (h)	Temperature (°C)	Seeds per Treatment	Germination (%)	Author
March	Growth chamber	No	12/12	20.5	100	67 to 97	[44]
September					150	50 to 90	
November	Greenhouse	No	Natural sunlight	21–24	10	66.1	[45]
December	Greenhouse	No	Natural sunlight	Ambient	25	90.67 to 91.38	[46]
October	Laboratory	Seeds floating on water up to 5 months	Fluorescent lights	22–25	50	94.4	[47]
		Seeds in leaf litter under forest canopy up to 5 months + 4 °C for 5 weeks in moist sand	Fluorescent lights	22–25	50	78.9	
January	Greenhouse	No	Natural sunlight	15–20	250	52.7	[48]
	Greenhouse	Floating seeds in water for	Natural sunlight	15–20	250		
		3 days				86.8	
		10 days				58.8	
		20 days				32.4	
	Greenhouse	Submerged seeds in water for	Natural sunlight	15–20	250		
		3 days				67.7	
		10 days				35.3	
		20 days				30.7	
Not defined	Field	Yes	Sunlight limited at 100, 65, 35, 7%	12.9–46.9	96	* ≈ 80	[49]
Not defined	Greenhouse	0–1.5 °C (no time defined) + water soaked for 48 h + 3–5 °C moist vermiculite for 3 months +	12/12	20–30	45		[50]
		Bare soil or				≈ 70	
		low leaf litter or				≈ 80	
		high leaf litter				≈ 50	
October	Growth chamber	Stratification for 8 weeks	Not defined	25	20	60.2	[51]
October	Growth chamber	Stratification for 8 weeks	Not defined	25	20	58.3	[52]
	Growth chamber	Gibberellic acid 40 ppm	Light/dark	30		good	[34]
Not defined	Growth chamber	Stored from 2 to 4 years + 4 °C for one month + Gibberellic acid 500 ppm	Dark	40 °C for 24 h	Not defined	good	[35]
October	Greenhouse	Seeds incubated under field conditions for 1 to 5 years at:		26.5	30–50		[38]
		soil depth = 10 cm	Natural sunlight			1.9 to 81	
		soil depth = 0 cm				79.4 to 83	
	Greenhouse	1–4 °C for 88 days	Natural sunlight	25/20	50	87	
	Greenhouse	Stored 5 years in lab conditions	Natural sunlight	Not defined	50	83.5	
Late summer	Greenhouse	1.7 °C for 28 days	Natural sunlight	Ambient	depending on source: 40, 43, or 64	0 to 78.1	[7]
December	Laboratory	Seeds incubated under litter and duff layers in field conditions	12/12 Fluorescent lights	18-20	100	28 and 79	[32]
December	Field	1 year with cold moist sand	High flux of sunlight Low flux of sunlight	Ambient	50	≈ 25 ≈ 21	[43]
		3 months with cold moist sand	High flux of sunlight Low flux of sunlight			≈ 15 ≈ 8	
October	Growth chamber	2 months at 17–20 °C	16/8	15	80	≈ 55	[37]
				20		≈ 25	
				30		≈ 18	
	Growth chamber	Stored 1 year (no treatment)	16/8	20	100	12	
		Stored 2 years (no treatment)				19	
		Stored 3 years (no treatment)				20	
	Field	no	Natural sunlight	Ambient	792	≈25	
Dispersal season	Growth chamber	4 °C more than 1 year	16/8	24/16	125	50.8	[41]
Fall	Growth chamber	4 °C during winter	Not defined	Not defined	Not defined	87	[36]
	Field		Natural sunlight	Ambient	100	≈ 6 to 9	
					80	≈ 6 to 13	
October	Growth chamber	No	12/12	15/6	25	0	[39]
				20/10		71	
				30/20		87	
			24 Dark	15/6		0	
				20/10		75	
				30/20		84	
	Growth chamber	4 °C for one month	12/12	15/6		19	
				20/10		51	
				30/20		82	
			24 Dark	15/6		7	
				20/10		37	
				30/20		89	
October	Growth chamber	Moist at 5 °C for 12 days	Dark	20(16 h)/30(8 h)	30	95	[42]
		Dry at 5 °C for 12 days				76	
		Moist at 25 °C for 12 days				84	
		Dry at 25 °C for 12 days				75	
		Control				70	
		5 °C for 4 days				77	
		5 °C for 12 days				96	
November	Growth chamber	4 °C for 1 month	Dark	25 25/30 30 40	100 naked embryos	40 73 94 51	[40]
Not defined	Growth chamber	Control	Not defined	20	100	26	[33]
		Sulfuric acid 95% for 10 min				20	
		Sulfuric acid 50% for 10 min				60	
		Hot water 95 °C for 15 min				40	
		3 °C for 10 days				29	
		3 °C for 15 days				32	
		3 °C for 20 days				52	

## Data Availability

The data presented in this study are available on request from the corresponding author.
